# Circadian Rhythms in Bacterial Sepsis Pathology: What We Know and What We Should Know

**DOI:** 10.3389/fcimb.2021.773181

**Published:** 2021-12-09

**Authors:** Malena Lis Mul Fedele, Camila Agustina Senna, Ignacio Aiello, Diego Andres Golombek, Natalia Paladino

**Affiliations:** ^1^ Laboratorio de Cronofisiología, Instituto de Investigaciones Biomédicas/Pontificia Universidad Católica Argentina - Consejo Nacional de Investigaciones Científicas y Técnicas (UCA-CONICET), Buenos Aires, Argentina; ^2^ Laboratorio de Cronobiología, Departamento de Ciencia y Tecnología, Universidad Nacional de Quilmes/Consejo Nacional de Investigaciones Científicas y Técnicas (CONICET), Buenos Aires, Argentina

**Keywords:** sepsis, circadian rhythms, immune system, hypothermia, infection

## Abstract

Sepsis is a syndrome caused by a deregulated host response to infection, representing the primary cause of death from infection. In animal models, the mortality rate is strongly dependent on the time of sepsis induction, suggesting a main role of the circadian system. In patients undergoing sepsis, deregulated circadian rhythms have also been reported. Here we review data related to the timing of sepsis induction to further understand the different outcomes observed both in patients and in animal models. The magnitude of immune activation as well as the hypothermic response correlated with the time of the worst prognosis. The different outcomes seem to be dependent on the expression of the clock gene Bmal1 in the liver and in myeloid immune cells. The understanding of the role of the circadian system in sepsis pathology could be an important tool to improve patient therapies.

## Introduction

### Sepsis and Septic Shock Characteristics

Sepsis is a syndrome characterized by multi-organ dysfunction caused by a deregulated host response to a pathogen, and is the primary cause of death from infection (Singer, 2016) affecting more than 30 million people worldwide every year with an in-hospital mortality rate of about 25-40% (Vincent et al., 2014; Acuna-Castroviejo et al., 2017; Huang et al., 2019). Sepsis symptoms vary according to the site of infection, the type of invasive microorganism, previous patient health and the time of detection; and include: body temperature alterations (fever or hypothermia), elevated heart and respiratory rate, altered mental state, glucose metabolism, inflammatory and hemodynamic variables, among others (Angus and van der Poll, 2013). Septic shock is defined as the septic condition worsened by metabolic and circulatory alterations, as hypotension, which increases the mortality rate (Singer, 2016). Patients who survive can present long-term physical, psychological, and cognitive disorders (Huang et al., 2019). Despite the severity of this pathology, there is no effective therapy that significantly reduces mortality and morbidity (Acuna-Fernandez et al., 2020).

Pathogen infection triggers an important inflammatory response, inducing an increase of pro-inflammatory cytokine release, such as IL-6, IL-1β and TNF-α, known as “cytokine storm”, generating a systemic inflammatory response [SIRS (Chen et al., 2014)]. The inflammatory response is not limited to the site of infection. The increased levels of IL-6 induce the production of the C-reactive protein in the liver, an acute response protein with anticoagulant and antiapoptotic function (among others), that can be used as a potential septicemia biomarker predictor (Cinel and Opal, 2009). Furthermore, an anti-inflammatory response, including glucocorticoid (Marik, 2011), IL-10, IL-1 receptor antagonist (IL-1Ra) and TGF-β secretion (Marchant et al., 1994; Marie et al., 1996), is also elicited. An excess of inflammation can cause tissue damage while an excess of anti-inflammatory response can favor secondary infections (van der Poll and Opal, 2008). The levels of IL-10 correlate with a worst patient prognosis (Gogos et al., 2000).

As in other pathologies, the central nervous system participates in sepsis development, as evidenced by the increase in proinflammatory molecule levels in this tissue (Meneses et al., 2019). Vagus nerve activation triggers the production of acetylcholine (one of the main vagus neurotransmitters), which inhibits synthesis and release of immune mediators by macrophages, such as TNF-α, increasing lifespan in septic mice (Borovikova et al., 2000; Andersson and Tracey, 2012). Moreover, the immune response activates the hypothalamic-pituitary-adrenal (HPA) axis which ends in glucocorticoids secretion, that modulates many body functions such as liver gluconeogenesis, glucose uptake, immune functions, among others (Kasahara and Inoue, 2015). Finally, the proinflammatory response can alter the blood-brain barrier (BBB), increasing its permeability (Danielski et al., 2018) and allowing the entrance of cytokines and leukocytes (Bohatschek et al., 2001; Hofer et al., 2008).

### Animal Models of Sepsis and Septic Shock

One of the most studied animal models of sepsis is induced by the administration of high doses (close to 20 mg/kg) of the bacterial endotoxin lipopolysaccharide (LPS), inducing high mortality rates (Liao and Lin, 2015; Ramos-Benitez et al., 2018) and the characteristic signs of sepsis (Nautiyal et al., 2009; Chuaiphichai et al., 2016; Cordoba-Moreno et al., 2018; Ramos-Benitez et al., 2018). It is a simple and reproducible technique, whose dose-dependent effects depend on the bacterial and mice strain used.

Administration of live bacteria is an easy, reproducible and low invasive method that can be used to study the response to a specific pathogen without surgery (Korneev, 2019). This technique can be useful to model hospital infections, which are often caused by monoinfections.

Cecal ligation and puncture (CLP) is considered a model of human appendix rupture or perforated diverticulitis. It is induced by midline laparotomy, exteriorization and ligation of the caecum, and puncture of the ligated caecum. The severity of the response can be adjusted by the size and number of punctions (Buras et al., 2005).

## Circadian Modulation of Septic Pathology

### Circadian Variations in Animal Models of Sepsis

There is a daily variation in the mortality rate due to septic shock: mice injected intraperitoneally with high doses of LPS at the end of their resting phase of activity (i.e., the end of the day) show a higher mortality rate (80% approximately) than those injected in the middle of the active phase of activity [the middle of the night, 30% approximately (Halberg et al., 1960; Marpegan et al., 2009)]. Similar results were obtained when TNF-α was administered intravenously (Hrushesky et al., 1994). Moreover, the clearance of Salmonella enterica was higher if the infection occurred during the active phase [the night (Bellet et al., 2013)]. Additionally, sepsis induced by CLP has a worse outcome when surgery is performed at the end of the active phase (the night) in comparison with the middle of the rest phase [the day (Heipertz et al., 2018)]. The differences in the time of poor prognosis between models could be related with differences in the kinetics of each stimulation method.

The relationship between sepsis and the molecular circadian clock machinery has also been studied. The molecular mechanism of the circadian clock arises from negative transcriptional feedback loops. The core loop includes the positive elements Clock and Bmal1, inducing the expression of the negative elements Per1-3 and Cry1-2, which, in turn, repress the transcriptional activity of the positive elements generating oscillations with periods close to 24 hours (Partch et al., 2014). It was shown that mice deficient for Per2 (Per2 KO) are more resistant to LPS-induced septic shock, showing lower levels of IFN-γ, IL-1β (Liu et al., 2006) and higher levels of glucocorticoids (Wang et al., 2015). There are many studies showing that the oscillation of circadian clock genes is disrupted in these mice (Zheng et al., 1999; Liu et al., 2006). Moreover, they also exhibited higher mRNA expression of the clock genes Clock and Bmal1 and of the enzyme Star, which participates in the adrenal synthesis of glucocorticoids, after LPS administration (Wang et al., 2015). Similarly, Clock-deficient mice showed greater survival after CLP-induced sepsis. Additionally, both mutants lost the daily difference in the mortality rate (Liu et al., 2006; Wang et al., 2016; Heipertz et al., 2018). Thus, it was suggested that the adrenal peripheral clock may play an important role in modulating the timing of LPS-corticoid secretion *via* regulation of the enzyme Star (Wang et al., 2015). Collectively, these data suggest that the daily differences observed in the mortality rate depend on the functioning of the clock rather than a specific clock component, since Per2 KO mice showed higher levels of Clock and both mutant animals were more resistant to sepsis than WT mice.

### Immune-Mediated Circadian Regulation

We recently reported that LPS-induced sepsis at the end of the resting phase elicited higher levels of TNF-α in serum than animals inoculated at the active phase (Mul Fedele et al., 2020). In addition, TNFR1-deficient mice not only had higher survival [which was previously observed by (Pfeffer et al., 1993; Leon et al., 1998; Guo et al., 2009)], but also abolished the daily difference. Additionally, it was observed that circadian variation in the mortality rate was abolished in mice lacking Toll-Like Receptor (TLR)-2 in sepsis induced *via* CLP (Heipertz et al., 2018).

Macrophages have an essential role during all sepsis stages (Freudenberg et al., 1986; Cheng et al., 2018). Peritoneal macrophages, the first ones to be activated after intraperitoneal stimulation, can be classified, according to their phenotype and function, in large and small cells, LPMs and SPMs, respectively. SPMs and LPMs exhibit specialized functions: SPMs present a pro-inflammatory functional profile, and LPMs appear to have a role in the maintenance of peritoneal cavity physiological conditions. LPMs are the most abundant subset of macrophages in unstimulated conditions, whereas SPMs are the minor subset. Nevertheless, in response to infectious or inflammatory stimuli, the cellular composition of the peritoneal cavity is altered (Cassado Ados et al., 2015). Despite the relatively little information focused on the functional profile induced by stimulation of SPM and LPM cells, data showed that both cell types can differentiate into both M1 (classically activated, pro-inflammatory) and M2 (alternative activated, anti-inflammatory) cells depending on the stimuli (Yuan et al., 2017; Li et al., 2021). Moreover, immune stimuli induce a decrease in LPMs levels, a phenomenon known as macrophage disappearance reaction [MDR (Okabe and Medzhitov, 2014)], which can include cell apoptosis (Luan et al., 2015), local clot form (Zhang et al., 2019) and migration (Okabe and Medzhitov, 2014). These cells accumulate in the omentum and can interact with mesothelial cells (Okabe and Medzhitov, 2014). Interestingly, this phenomenon was more pronounced after LPS administration at the time of higher mortality [at the end of the resting phase (Mul Fedele et al., 2020)]. In response to a sterile injury in the liver, LPMs invaded afflicted tissue *via* direct recruitment across the mesothelium (Wang and Kubes, 2016). Regarding this, it was recently found that conditional deletion of Bmal1 in hepatocytes (Bmal1^ΔHep^) results in constitutively high LPS sensitivity (Geiger et al., 2021) suggesting that liver signals could induce the arrival of LPMs in a clock-dependent way.

The decrease in the levels of LPMs is accompanied by the arrival of inflammatory monocytes (Cassado Ados et al., 2015) which can differentiate into SPMs (Ghosn et al., 2010), which increase 2 days after LPS stimulation. These cells showed higher levels at the end of the resting phase (Mul Fedele et al., 2020). *In vitro* studies have shown that SPMs develop a pro-inflammatory profile in response to LPS (Cain et al., 2013) suggesting a relation with the higher levels of TNF-α observed at this time (Mul Fedele et al., 2020).

Spleen macrophages are also activated (measured by CD86 expression) in response to LPS (Liu et al., 2006). Interestingly, this activation increases after LPS administration during the resting phase, but not in the active phase (Mul Fedele et al., 2020).

Recent works showed that conditional deletion of Bmal1 in myeloid cells (Bmal1^ΔMye^), which disrupt the macrophage clock, accelerated death in sepsis induced by both CLP (Deng et al., 2018) or LPS (Curtis et al., 2015) and abolished the daily differences in the mortality rate. Bmal1^ΔMye^ mice developed higher pro-inflammatory (Curtis et al., 2015) and anti-inflammatory responses (Deng et al., 2018). The interaction between the pathogen and the host immune system is very complex and comprises two stages: an early stage characterized by excessive inflammation and a late stage characterized by sustained immune suppression (van der Poll et al., 2017). Therefore, it is not surprising to find that Bmal1 is related to pro-inflammatory and anti-inflammatory processes, which are both deregulated during sepsis. In contrast, daily differences after LPS inoculation were not abolished in Bmal1^ΔMye^ mice maintained under a time-restricted feeding schedule (Geiger et al., 2021). This discrepancy could be due to the different kinetics observed in the sepsis-induced mortality between models since the survival time was shorter in the last mentioned study. In addition, it is possible that mice fed only during the night have more robust peripheral circadian rhythms that may compensate for the lack of Bmal1 in myeloid cells, similar to what happens with the rhythm of the respiratory exchange rate in the same work (Geiger et al., 2021).

Regarding anti-inflammatory mechanisms, the serum levels of IL-10 and corticosterone increased after LPS injection, without time differences (Mul Fedele et al., 2020; Geiger et al., 2021). It has been shown that, in animal models, the stimulation of the HPA axis (which is responsible for glucocorticoid secretion) increases resistance to the endotoxic shock (Dejager et al., 2010; Gilibert et al., 2014). However, clinical therapy with glucocorticoids does not always induce longer survival and can generate treatment resistance (Dendoncker and Libert, 2017). Additionally, melatonin, which participates in circadian rhythm regulation, has anti-inflammatory properties and protects against sepsis-induced cardiac (Zhang et al., 2019), lung (Li et al., 2020), liver (Chen et al., 2019) and renal dysfunction (Dai et al., 2019). These effects could be dependent on melatonin receptor-induced neutrophil activity (Xu et al., 2019).

This evidence may indicate that circadian control over some physiological functions, such as the immune system, that are altered during sepsis, can have an important role on the pathogenesis of this syndrome.

### Circadian Regulation of Body Temperature During Sepsis

As previously mentioned, another feature of sepsis is the alteration of body temperature: fever or hypothermia, the latter being associated with a worse prognosis (Angus and van der Poll, 2013) and higher inflammation (Nautiyal et al., 2009; Stewart et al., 2010; Garami et al., 2018). Interestingly, in both CLP (Silver et al., 2012) and LPS-induced sepsis (Mul Fedele et al., 2020) deeper hypothermia correlated with higher mortality (the active phase and the end of the resting phase, respectively).

The hypothalamic preoptic area (POA) is the main integrative brain site for thermoregulation (Morrison and Nakamura, 2018), but also the suprachiasmatic (SCN) and paraventricular (PVN) nucleus modulate thermal signals (Wanner et al., 2013; Guzman-Ruiz et al., 2015). LPS inoculation induces neuronal activation of these brain regions (Hare et al., 1995; Marpegan et al., 2005; Paladino et al., 2014). Interestingly, this activation (measured by cFOS expression) was increased after LPS inoculation at the time of higher mortality rate [the end of the resting phase (Mul Fedele et al., 2020)]. TNF-α and TNFR1 participate both in the immune-circadian communication (Cavadini et al., 2007; Duhart et al., 2013; Paladino et al., 2014) and in the hypothermic response to LPS (Leon et al., 1998; Nautiyal et al., 2009). We have shown that these molecules were induced in the POA in response to septic shock, although it was independent of the time of LPS inoculation (Mul Fedele et al., 2020).

Temperature is a well-known circadian entraining cue for peripheral oscillators. It was shown that circadian changes in temperature, similar to that seen in core temperature rhythms, can entrain and enhance the amplitude of circadian rhythms in the periphery (Buhr et al., 2010). In addition, the circadian clock is responsible for the development of a body temperature circadian rhythm (Refinetti and Menaker, 1992), so that this bidirectional interaction is disrupted during sepsis.

### Role of the Circadian System in Metabolic and Circulatory Alterations

There are many interactions between metabolic or circulatory functions and the clock machinery. As previously mentioned, septic patients can develop hypoglycemia through the induction of glycolysis, which could be lethal if it is not compensated with liver gluconeogenesis (Weis et al., 2017). Using a time-restricted feeding protocol, the LPS-induced hypoglycemia correlated with higher mortality rate [the end of the resting phase (Geiger et al., 2021)], while β-hydroxybutyrate levels, which induces NLRP3 inflammasome (Youm et al., 2015), are increased at this time. This inflammasome, along with reactive oxygen and nitrogen species and NF-κB pathway can be regulated in the mitochondria both by clock genes and melatonin [reviewed in (Acuna-Castroviejo et al., 2017)].

The circadian clock also regulates coagulation and fibrinolysis processes (Budkowska et al., 2019). Bmal1-deficient mice exhibited a hypercoagulable state and an enhanced arterial and venous thrombogenicity (Hemmeryckx et al., 2019). Moreover, the lack of Bmal1 induces alterations in the levels of coagulation factors, contributing to a coagulation abnormality in *S. oralis* infection (Chen et al., 2020). In addition, tissue factor (TF), which participates in coagulation and in LPS-induced inflammation and mortality, is under circadian regulation in the liver (Oishi et al., 2013).

In spite of these data, it remains to be explored what happens with these mechanisms when sepsis is induced at different times of the day and what happens with their circadian rhythms during the pathology.

### Sepsis and Circadian Desynchronization

Desynchronization of circadian rhythms achieved by different protocols (SCN injury or chronic jet-lag models) increased LPS-induced inflammation (Castanon-Cervantes et al., 2010; Adams et al., 2013; Guerrero-Vargas et al., 2014), while constant light conditions (also disruptive) reduced the survival rate after CLP (Carlson and Chiu, 2008). Interestingly, the exacerbation in the mortality rate and hypothermic response is accompanied by an increase in the TNF-α levels in desynchronized mice (Mul Fedele et al., 2020). Similarly, four inversions of the LD cycle (1 per week) increased the mortality rate (Castanon-Cervantes et al., 2010). Moreover, other studies from our group have shown that in constant dark conditions, the daily difference in the response to LPS is lost with low survival percentages at all times (Marpegan et al., 2009). This suggests that the absence of temporal external cues as well as desynchronization can reduce the survival percentage in sepsis. This data also supports the bidirectional communication between the immune system and the circadian system (Marpegan et al., 2009) and suggests a strong influence of the central clock on susceptibility to immune stimuli.

Additionally, the uncoupling of central and peripheral clocks induced by reversed feeding (feeding only at the resting phase) also increased the mortality rate and exacerbated the inflammatory response, compared with active phase-fed mice, after CLP (Oyama et al., 2014). Interestingly, reversed feeding inverted the time of higher mortality induced by LPS inoculation (Geiger et al., 2021). In addition Bmal1^ΔHep^ mice developed constitutively high LPS sensitivity independently from light or feeding schedules, in a farnesoid X receptor (FXR)-dependent way (Geiger et al., 2021).

### Influence of the Circadian System in Patient Response

In relation to patients, it has been shown that *Salmonella abortus* inoculation at the end of the active phase (the day) induces higher levels of serum cortisol, compared to its administration at the beginning of the active phase [the day (Pollmacher et al., 1996)]. In addition, a day-night difference in the acute phase response to endotoxemia exists in healthy volunteers with a more pronounced inflammatory response during the resting phase [the night (Alamili et al., 2014)]. Moreover, septic patients had higher levels of cytokines than other intensive care unit (ICU) patients (Acuna-Fernandez et al., 2020) and exhibited several alterations in clock gene rhythms (Coiffard et al., 2019; Acuna-Fernandez et al., 2020; Lachmann et al., 2021). These alterations were accompanied by an increase in urinary 6-sulfatoxymelatonin (aMT6s) excretion, which is related to chronodisruption (Acuna-Fernandez et al., 2020). A longitudinal study reported that individuals who developed septic shock during their ICU stay had an increase in aMT6s and a decrease in cortisol levels compared with entry and discharge from the ICU. Also, higher aMT6s or cortisol mean values were correlated with lower in-hospital mortality (Sertaridou et al., 2018). Studies carried out in patients who suffered severe trauma showed that the early alteration of the cortisol rhythms are associated with sepsis development. These parameters were accompanied by clock gene and leucocyte count alterations (Coiffard et al., 2019). However, changes in the rhythms of clock gene expression and rest-activity were also observed in non-septic ICU patients (Maas et al., 2020a; Maas et al., 2020b). Since sleep deprivation increases inflammation (Shearer et al., 2001; Frey et al., 2007), it is relevant to consider that ICU normally have constant light conditions, which can complicate the inflammatory response.

Because of the correlation between sepsis and circadian rhythms, chronobiological therapies have been proposed. As septic patients have shown alterations in circadian rhythms and sleep in ICU, a few studies used light therapy to entrain circadian rhythms. In septic animals, exposure to bright blue light enhanced bacterial clearance, reduced systemic inflammation and organ injury (Lewis et al., 2018). However, light therapy did not improve patient prognosis, although these works used low intensity light [reviewed in (Jacob et al., 2020)]. As previously mentioned, melatonin has shown anti-inflammatory properties in sepsis (Marra et al., 2019; Jacob et al., 2020). In addition, melatonin reduced IL-1β and YKL-40 plasma levels and oxidative stress response in a human model of endotoxemia during the active phase, but not during the resting phase (Alamili et al., 2014).

## Conclusion

**Figure 1:**
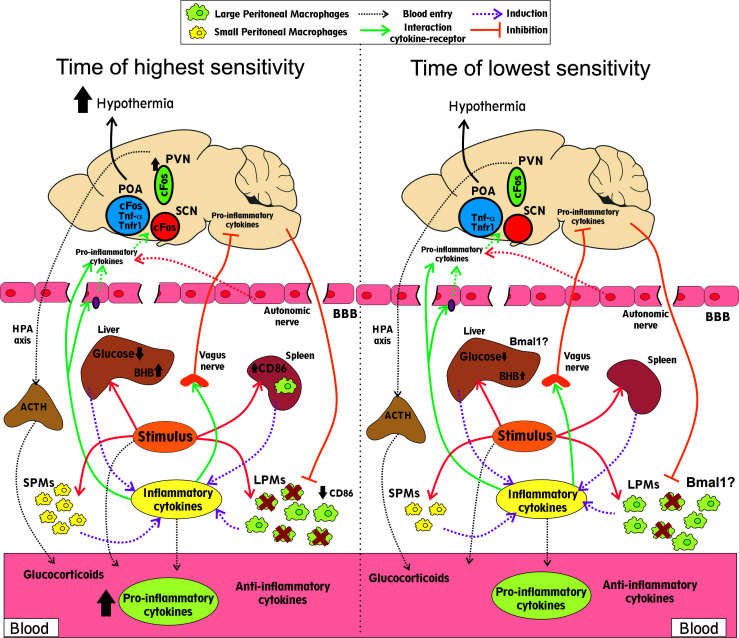
Differential features observed at times of higher and lower mortality rates. Infectious stimuli impact the peritoneal macrophages (SPMs and LPMs), in the liver and the spleen, secreting higher cytokine levels to the blood at the time of higher mortality. This difference could be related to the higher basal SPMs counts and higher activated (CD86) splenic macrophages after stimulation. The decrease in the LPMs count (MDR) is also higher at the time of the worst prognosis. The anti-inflammatory mediators glucocorticoids and IL10 increase similarly at both times. The decrease in glucose and the increase in β-hydroxybutyrate (BHB) levels were more pronounced at the moment of higher mortality and depending on the liver rhythms. Neuronal activation (cFos) of brain regions that control body temperature (POA, SCN and PVN), and the subsequent hypothermia, were also increased at the time of poor prognosis. Communication between the central nervous system and peripheral tissues during sepsis includes the HPA axis and the autonomic nerves, particularly the vagus nerve. The brain blood barrier (BBB) can receive inflammatory signals and secrete cytokines into the brain. In addition, the inflammation that occurs during sepsis can disturb this barrier and increase its permeability. The expression of the clock gene Bmal1 in the liver and the myeloid cells is associated with a better prognosis.

Despite the numerous evidences that show a circadian component of the septic response, the causes underlying this differential response are still unknown. **Figure 1** summarizes the main findings that could explain the different outcomes after sepsis induction at different times. Immune stimuli induce higher cytokine secretion at the time of higher mortality (the end of the resting phase), which could be secreted by SPMs or splenic macrophages, among other cells. In addition, the MDR is higher at the time of worse prognosis (the end of the resting phase). Circadian rhythms in the liver could be relevant for the increase in glucose metabolism alterations and acute phase protein secretion, including pro-inflammatory cytokines, at the moment of higher mortality. In addition, activation of brain regions that control body temperature, and the subsequent hypothermia, were also associated with poor prognosis (the end of the resting phase). Lastly, the levels of glucocorticoids and IL10, both anti-inflammatory mediators, increase similarly at different times. The nervous system, particularly the HPA axis and autonomic nerves, regulates many of these functions. To further understand the causes of the daily differences in sepsis prognosis it will be relevant to deepen the knowledge of the mechanisms inducing a higher pro-inflammatory immune response at specific times and, also, to analyze the role of the nervous system and the liver at each time of day.

The understanding of the role of the circadian clock in patients in the ICU, particularly after infections or severe trauma, could help to improve sepsis treatments. In addition, circadian evaluation could be used as a prognosis marker. In this regard, it will be useful to measure rhythmic variables, like immune cell count, melatonin or cortisol, at different times of the day, during the early stage of the disease in order to associate this with patient prognosis. Likewise, analyzing the time of infection, for example in individuals who suffered severe trauma, would allow us to assess whether the circadian differences observed in animal models can be extrapolated to humans.

## Author Contributions

MM and NP initiated and designed the overall concept and wrote the manuscript. CS and IA wrote the manuscript. All authors revised the manuscript, approved the final version and approved it for publication.

## Funding

This work was supported by grants from the National Research Council (CONICET), and the National University of Quilmes (UNQ).

## Conflict of Interest

The authors declare that the research was conducted in the absence of any commercial or financial relationships that could be construed as a potential conflict of interest.

## Publisher’s Note

All claims expressed in this article are solely those of the authors and do not necessarily represent those of their affiliated organizations, or those of the publisher, the editors and the reviewers. Any product that may be evaluated in this article, or claim that may be made by its manufacturer, is not guaranteed or endorsed by the publisher.
